# 30% Supramolecular Salicylic Acid Improved Symptoms and Skin Barrier in Papulopustular Rosacea

**DOI:** 10.1111/jocd.70046

**Published:** 2025-02-19

**Authors:** Zihan Wang, Yutong Wu, Fatemeh Nozzari Varkani, Xian Su, Ziping Yang, Xinghua Gao, Li Zhang

**Affiliations:** ^1^ Department of Dermatology The First Hospital of China Medical University Shenyang Liaoning China; ^2^ Department of Medical Cosmetic Shenzhen Nanshan People's Hospital Shenzhen Guangdong China; ^3^ Department of Dermatology Children's Hospital of Soochow University Suzhou Jiangsu China

**Keywords:** chemical peeling, papulopustular rosacea, skin barrier, supramolecular salicylic acid

## Abstract

**Background:**

Rosacea is a chronic skin condition that affects millions of people worldwide, and its management continues to pose a significant challenge in dermatology.

**Aim:**

In this study, we investigated the efficacy and safety of 30% supramolecular salicylic acid (SSA) as a treatment for papulopustular rosacea, with a particular focus on improving skin lesions, reducing persistent erythema, and enhancing skin barrier function.

**Methods:**

Thirty‐four patients diagnosed with papulopustular rosacea were randomly divided into an experimental group treated with 30% SSA and a control group receiving a placebo. Clinical outcomes were evaluated based on lesion reduction rates, investigator severity assessment (ISA) scores, VISIA red area scores, and skin barrier, including trans‐epidermal water loss (TEWL), sebum levels, stratum corneum hydration, and pH values.

**Results:**

A total of 34 patients were collected for both the experimental and control groups, with no statistical differences in age or disease severity between the groups (*p* > 0.05). The effective rate was 68.75% in the experimental group (*p* < 0.01). After treatment, the ISA score was 1.75 ± 0.68 in the experimental group and 2.40 ± 0.83 in the control group, indicating significant improvement (*p* < 0.05). The improvement rate of the VISIA redness score was 25.1% in the experimental group (*p* < 0.01). Among the 17 patients who underwent skin barrier function testing. Skin hydration significantly improved on left cheek (*p* < 0.05), right cheek (*p* < 0.01), and nose (*p* < 0.05) after 30% SSA treatment in experimental group. Sebum levels were significantly reduced on both cheeks and forehead (*p* < 0.05). No statistical differences were observed in other locations. Skin TEWL and pH value showed no changes.

**Conclusion:**

30% SSA reduced papules and pustules in rosacea, improved persistent erythema, and enhanced stratum corneum hydration in the 30% SSA‐treated group compared to the placebo group. No significant differences were observed in TEWL and skin pH values between the two groups. Our findings suggest that 30% SSA is an effective and safe option for managing papulopustular rosacea, offering a well‐tolerated alternative to traditional treatments.

## 1 Introduction

Rosacea is a chronic inflammatory skin condition [1] primarily affecting the central face [2], characterized by persistent erythema, flushing, telangiectasia, and sometimes accompanied by sensations of burning or stinging [3]. While topical and oral medications [4] are commonly used to manage symptoms and reduce flare‐ups, rosacea can significantly impact patients' quality of life. The condition is classified into four main subtypes: erythematotelangiectatic, papulopustular, phymatous, and ocular rosacea [5]. Among these, papulopustular rosacea (PPR) is more prevalent and is often associated with impaired skin barrier function [6].

The skin barrier, which refers to the outermost protective layer of the skin [7, 8], plays a critical role in preventing water loss and external irritants. Its integrity can be assessed using several parameters, including trans‐epidermal water loss (TEWL), stratum corneum hydration, sebum content, and skin pH value. Maintaining skin barrier function is crucial for overall skin health, and therapies targeting skin barrier repair are essential in the management of rosacea [9].

Traditional treatments, including topical and systemic administration antibiotics, retinoid, andβ‐blocker [3], are often associated with potential side effects such as allergic reactions, gastrointestinal discomfort, or liver function impairment [4, 10]. Laser treatments can be an effective option, but the prolonged treatment duration leads to substantial expenses. Chemical peeling is another widely used treatment in dermatology to improve various skin conditions by removing the outermost layers of epidermis [5, 11], The peeling agents are typically composed of acidic substances, such as alpha‐hydroxy acids (AHAs), azelaic acid, acetic acid, and salicylic acid. Salicylic acid (SA) is a beta‐hydroxy acid (BHA) [12], posessing high permeability, as well as anti‐inflammatory and antimicrobial properties [13, 14], which makes it an effective treatment for many skin conditions, including rosacea.

Supramolecular salicylic acid (SSA) is a novel formulation that uses supramolecular technology to covalently bond salicylic acid with poloxamer 407 [15, 16], forming a more stable compound and easily dissolves in water. Despite its potential, research on the use of 30% SSA for rosacea treatment remains limited, with most studies focusing on combination therapies [1, 17, 18]. There is a lack of data on the independent therapeutic effects of 30% SSA. Therefore, this study aims to investigate the efficacy of 30% SSA as a standalone treatment for PPR, particularly in improving skin lesions, reducing erythema, and enhancing skin barrier function. By providing more robust clinical evidence, this study seeks to establish SSA as a viable and effective option for managing rosacea.

## 2 Materials and Methods

Thirty‐four patients with PPR, the primary phenotype, who sought treatment at the dermatology outpatient clinic of our hospital between July 2020 and December 2020, were recruited for this study. All patients signed informed consent forms prior to enrollment. This study was conducted following approval by the Ethics Committee of the First Hospital of China Medical University.

### 2.1 Patient Selection

*Inclusion criteria*: (1) Patients aged from 18 to 60 years and (2) diagnosed with PPR as the primary phenotype, based on the latest diagnostic criteria from the National Rosacea Society (2017 edition) [19].

*Exclusion criteria*: (1) Patients with other facial skin conditions that could interfere with study outcomes, such as melasma, freckles, or seborrheic dermatitis; (2) patients who had taken oral retinoids within 2 months prior to the study (6 months for acitretin) or used topical tacrolimus ointment within 3 months; patients with a history of facial injections or surgery within 2 months; (3) pregnant, planning to become pregnant, or breastfeeding patients; (4) patients with concurrent acne at the time of the study or those with chronic illnesses requiring long‐term treatment; (5) patients with a history of drug intolerance or undergoing anti‐allergy treatment; patients with immune system disorders or receiving immunosuppressive therapy; (6) patients who had used oral antibiotics (e.g., minocycline and doxycycline) or adrenergic blockers (e.g., carvedilol) within 4 weeks prior to the study; patients using topical rosacea treatments (e.g., fusidic acid, azelaic acid, and brimonidine), or who had undergone physical treatments such as chemical peels (e.g., salicylic acid or alpha hydroxy acids) within 4 weeks; and (7) patients with known hypersensitivity to the study medications.

### 2.2 Study Design and Treatment

The objective of this study is to evaluate the efficacy and safety of 30% supramolecular salicylic acid used alone. Patients were randomly assigned to either the experimental group or the control group. The experimental group received treatment with 30% SSA (Broda, Borenda Biochemical Technology, Shanghai, China) once every 2 weeks for a total of four treatments. The control group received a salicylic acid placebo (comprising the vehicle matrix of the supramolecular salicylic acid but without the active salicylic acid ingredient, Broda, Borenda Biochemical Technology, Shanghai, China).

During each visit, all patients underwent facial photography using the VISIA skin analysis system, and measurements were taken for TEWL (Tewameter TM300, Courage Khazaka, Germany), sebum levels (Sebumeter SM815‐MPA, Courage Khazaka, Germany), stratum corneum hydration (Corneometer CM825, Courage Khazaka, Germany), and skin pH, (Skin‐PH‐Meter PH905, Courage Khazaka, Germany). After skin examination, 30% SSA were applied on the whole face of patients by massaging with repeatedly brushing water. The whole procedure lasted for 10–15 min. A cold compression (Broda, Borenda Biochemical Technology, Shanghai, China). was used after each treatment to help sedation.

All participants were invited to undergo skin barrier tests indoors, away from direct sunlight and wind. The testing environment was maintained at a temperature of 22°C–25°C and humidity of 40%–60%. Participants were instructed not to wash their faces with water within 3 h prior to the test and to rest indoors for 20 min before testing. Subsequently, measurements of TEWL (transepidermal water loss), stratum corneum hydration, skin oiliness, and pH were taken sequentially. Measurement sites included both cheeks, nose, and forehead on both sides. Each measurement was conducted three times, and the average value was recorded.

### 2.3 Evaluation Methods for Efficacy

The evaluation involved counting the number of papules, pustules, and the total number of lesions (the sum of papules and pustules) for each patient. Additionally, VISIA red area scores, patient Investigator Severity Assessment (ISA) scores, and skin barrier function measurements were recorded.

After all treatments, the following metrics were calculated: lesion reduction rate, overall efficacy rate, and VISIA red area score improvement rate.

### 2.4 Lesion Reduction Rate

The lesion reduction rate was calculated as Lesion reduction rate = (Total lesions before treatment − Total lesions after treatment)/Total lesions before treatment × 100%.

It was classified into four categories: Complete resolution (lesion reduction rate ≥ 90%); Significant improvement (60% ≤ lesion reduction rate < 90%); Improvement (20% ≤ lesion reduction rate < 60%); No improvement (lesion reduction rate < 20%).

### 2.5 Overall Efficacy Rate

Overall efficacy rate = (Number of cases with significant improvement + Number of cases with complete resolution)/Total sample size × 100%.

### 2.6 ISA (Investigator Severity Assessment) Score

The severity of the condition was rated on a 0–4 scale [20]: None: No erythema, no papules/pustules = 0 points; Very mild: Very mild erythema, minimal papules/pustules = 1 point; Mild: Mild erythema, few small papules/pustules = 2 points; Moderate: Moderate erythema, moderate number of papules/pustules, papules/pustules may be larger = 3 points; Severe: Severe erythema, numerous large papules/pustules = 4 points.

### 2.7 VISIA Red Area Score

The VISIA red area score was collected form VISA skin analysis system [21]. And improvement rate was scored on a 0–3 scale: None (0), Mild (1), Moderate (2), Severe (3). Improvement rate = (VISIA red area score after treatment–VISIA red area score before treatment)/VISIA red area score before treatment × 100%.

### 2.8 Statistic Analysis

The data were analyzed using GraphPad Prism 8.0 software. Results were presented as mean ± standard deviation, and comparisons between group means were performed using ANOVA. Statistical significance was defined as *p* < 0.05, with *p* < 0.01 indicating a highly significant difference.

## 3 Results

### 3.1 Demographics

Totally, 34 patients were collected. Three patients were lost to follow‐up, as one developed a suspected allergy to the cold compression, and two were not satisfied with the treatment after the first visit. Finally, a total of 31 patients completed this study, including 16 patients in the experimental group, with a mean age of (37.56 ± 12.06) years, and 15 patients in the control group, with a mean age of (36.07 ± 10.17) years. There was no statistically significant difference in age between the two groups (*p* = 0.713).

The initial ISA score for the experimental group was (2.94 ± 0.68), and for the control group was (2.87 ± 0.92), showing no statistically significant difference (*p* = 0.808).

### 3.2 30% SSA Reduced ISA Score and Effectively Treated Rosacea

**FIGURE 1:**
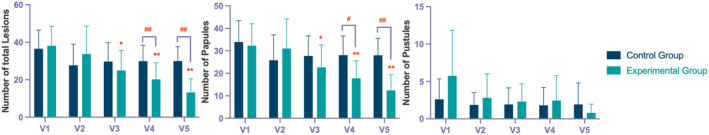
Numer of lesions variation. **Indicates a statistically significant decrease within the same group compared to V1 (*p < 0.05, **p < 0.01); #, ##Indicates a statistically significant difference between the experimental and control groups (#*p* < 0.05, ##*p* < 0.01).

All patients skin lesions were counted by two experienced physicians and calculated as papules and pustules (Tables 1 and 2 and Figure 1) No statistically significant difference between the experimental and control groups at first visit (*p* > 0.05). At the end of the experiment: 1 case showed complete remission of lesions, 10 cases showed significant improvement, 5 cases improved, and no cases were ineffective in the experimental group, resulting in an efficacy rate of 68.75%. In the control group, no cases achieved significant improvement, 6 cases showed moderate improvement, and 9 cases were ineffective, resulting in an efficacy rate of 0%. Fisher's exact test showed *p* < 0.01, indicating a statistically significant difference, suggesting that 30% SSA treatment is associated with the improvement of lesions in the study subjects.

**TABLE 1:** Efficiency and lesion reduction rate before and four treatments with 30% supramolecular salicylic acid (mean ± SD) and lesion reduction rate.

Lesion reduction rate									
	Numbers	Complete remission	Significant improvement	Improvement	No improvement	Efficiency (%)	Total lesions		*p*
							Before treatment (V1)	After final treatment (V5)	
Control Group	15	0	0	6	9	0	36.53 ± 9.96	29.93 ± 7.69	> 0.05
Experimental group	16	1	10	5	0	68.75	38.06 ± 10.40	13.19 ± 7.30^**,##^	< 0.01
							*p* > 0.05	*p* < 0.01	

*Note:* **Indicates a statistically significant difference within the same group compared to V1. ##Indicates a statistically significant difference between the experimental and control groups (*p* < 0.01).

**TABLE 2:** Changes in number of lesions before and after each treatment with 30% supramolecular salicylic acid (mean ± SD) of different kinds of lesions (total lesions, papules, and pustules).

Number of lesions (per person)						
Visits	Total		Papules		Pustules	
	Control group (*n* = 8)	Experimental group (*n* = 9)	Control group (*n* = 8)	Experimental group (*n* = 9)	Control group (*n* = 8)	Experimental group (*n* = 9)
V1	36.53 ± 9.96	38.06 ± 10.40	33.93 ± 9.59	32.31 ± 9.70	2.60 ± 2.74	5.75 ± 6.10
V2	27.67 ± 11.28	33.69 ± 14.92	25.80 ± 11.29	31.00 ± 13.18	1.87 ± 1.64	2.81 ± 3.22
V3	29.67 ± 10.15	24.94 ± 10.63**	27.73 ± 8.92	22.63 ± 9.98*	1.93 ± 2.21	2.31 ± 2.38
V4	29.87 ± 8.44	20.19 ± 8.84^**,##^	28.07 ± 8.58	17.75 ± 7.86^**,#^	1.80 ± 2.39	2.44 ± 3.36
V5	29.93 ± 7.69	13.19 ± 7.30^**,##^	28.00 ± 7.58	12.38 ± 6.97^**,##^	1.93 ± 2.86	0.81 ± 1.16

*Note:* *, **Indicates a statistically significant difference within the same group compared to V1 (*p < 0.05, **p < 0.01); #, ##Indicates a statistically significant difference between the experimental and control groups of each visit (#*p* < 0.05, ##*p* < 0.01).

**FIGURE 2:**
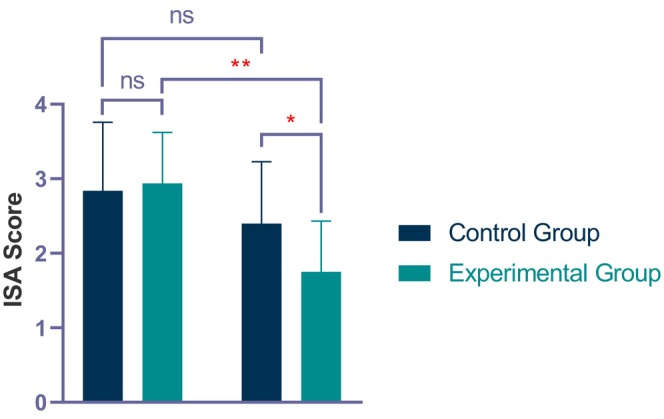
ISA score before and after 4 treatments. *p < 0.05; **p < 0.01.

At the initial visit (V1), the ISA score of the experimental group was 2.94 ± 0.68, while that of the control group was 2.87 ± 0.92, with *p* > 0.05, indicating no statistically significant difference in disease severity between the two groups before treatment (Table 3 and Figure 2). At the final visit (V5), ISA score in the experimental group (1.75 ± 0.68) significantly decreased (*p* < 0.01). Additionally, a statistically significant difference was found between the experimental and control groups at V5 (*p* < 0.05). No statistically significant difference in the ISA scores before and after treatment within the control group (*p* > 0.05).

**TABLE 3:** ISA score before and after 4 treatments (mean ± SD).

ISA score	Before treatment (V1)	After treatment (V5)	Overall *p* value
Control group	2.87 ± 0.92	2.40 ± 0.83	0.154
Experimental group	2.94 ± 0.68	1.75 ± 0.68^*,##^	0.000
Overall *p* value	0.808	0.024	

*Note:* *Indicates a statistically significant difference within the same group compared to V1 (*p < 0.05); ##Indicates a statistically significant difference between the experimental and control groups of each visit (##*p* < 0.01).

### 3.3 30% SSA Treatment Reduced VISIA Red Area Index

**FIGURE 3:**
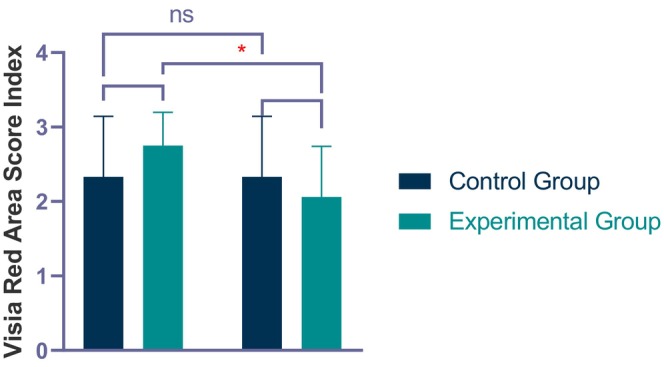
VISIA Red Area Score Index before and after 4 treatments. *p < 0.05.

Persistent erythema is one of the representative symptoms of rosacea patients. The VISIA red area score can be used to assess the severity of facial erythema in patients [21]. There was no statistically significant difference between the experimental and control groups at the first visit (V1, before treatment) (*p* > 0.05). A one‐way ANOVA showed that the VISIA redness score in the experimental group significantly decreased from the first to the final visit, with a statistically significant difference (*p* < 0.01) (Table 4 and Figure 3). The improvement rate of the VISIA redness score was 25.1% in the experimental group and 0% in the control group. Figure 3 shows the improvement in lesions and VISIA red area index over the course of four treatment sessions of one patient.

**TABLE 4:** Visia Red Area Score Index before and after 4 treatments (mean ± SD).

Visia red area score index	Improvement rate (%)	Before treatment (V1)	After treatment (V5)	Overall *p* value
Control group	0.00	2.33 ± 0.816	2.33 ± 0.816	1.000
Experimental group	25.10	2.75 ± 0.447	2.06 ± 0.68^#^	0.015
Overall *p* value		0.192	0.494	

*Note:* #Indicates a statistically significant difference before and after 4 treatments in the experimental group (*p* < 0.05).

### 3.4 30% SSA Treatment Improved Skin Barrier Function

In this study, skin barrier function was assessed in 17 patients (9 in the experimental group and 8 in the control group).

#### 3.4.1 TEWL Measurements in the Experimental Group

**FIGURE 4:**
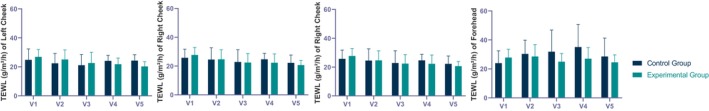
Changes of skin TEWL (transepidermal water loss) (g/cm^2^ h^−1^) before and after 4 treatments.

Trans‐epidermal water loss values on the cheeks of the experimental group showed a downward trend, though there was no statistically significant difference (Table 5 and Figure 4).

**TABLE 5:** Changes of skin TEWL (Transepidermal water loss) (g/cm^2^ h^−1^) before and after 4 treatments (mean ± SD).

TEWL (g/cm^2^ h^−1^)								
Visits	Left cheek		Right cheek		Nose		Forehead	
	Control group (*n* = 8)	Experimental group (*n* = 9)	Control group (*n* = 8)	Experimental group (*n* = 9)	Control group (*n* = 8)	Experimental group (*n* = 9)	Control group (*n* = 8)	Experimental group (*n* = 9)
V1	24.81 ± 7.59	26.89 ± 5.1	27.73 ± 5.15	25.7 ± 6.06	32.86 ± 7.40	36.51 ± 6.58	23.98 ± 8.52	27.84 ± 5.81
V2	22.43 ± 6.78	25.09 ± 6.58	24.68 ± 6.66	24.5 ± 8.23	35.01 ± 6.79	37.36 ± 11.11	30.34 ± 9.48	28.58 ± 8.21
V3	21.11 ± 7.4	22.6 ± 7.4	22.44 ± 6.25	22.83 ± 8.55	30.84 ± 6.06	33.63 ± 7.56	31.85 ± 14.97	24.96 ± 5.66
V4	24.14 ± 3.84	21.84 ± 4.2	22.28 ± 6.13	24.66 ± 4.22	29.81 ± 7.29	32.83 ± 12.82	35.06 ± 15.65	27.02 ± 7.7
V5	24.26 ± 4.11	20.29 ± 3.29	20.68 ± 3.27	22.21 ± 5.44	33.75 ± 13.01	29.24 ± 10.82	28.58 ± 12.68	24.52 ± 5.19

#### 3.4.2 Sebum Value Changes in the Experimental and Control Groups

**FIGURE 5:**
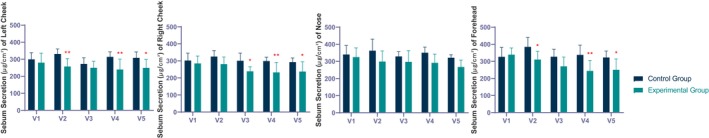
Changes of skin sebum before and after 4 treatments *, **Indicate a statistically significant difference within the same group compared to V1 (*p* < 0.05); **Indicates a statistically significant difference within the same group compared to V1. *p < 0.05; **p < 0.01.

Sebum levels on both cheeks and forehead in the experimental group were reduced at final visit (*p* > 0.05). At V2, V4, and V5, the sebum content of the left cheek and forehead in the experimental group was significantly lower than that in the control group (*p* < 0.05). On the right cheek, sebum levels were significantly lower in the experimental group compared to the control group at V3, V4, and V5 (*p* < 0.05). No significant statistical difference was observed in sebum values of the nose before and after treatment (Table 6 and Figure 5).

**TABLE 6:** Changes of skin sebum before and after 4 treatments (mean ± SD).

Sebum (μg/cm^2^)								
Visits	Left cheek		Right Cheek		Nose		Forehead	
	Control group (*n* = 8)	Experimental group (*n* = 9)	Control group (*n* = 8)	Experimental group (*n* = 9)	Control group (*n* = 8)	Experimental group (*n* = 9)	Control group (*n* = 8)	Experimental group (*n* = 9)
V1	299.25 ± 39.14	279.89 ± 55.90	302 ± 43.67	285 ± 43.56	340 ± 52.97	325.33 ± 53.82	326.63 ± 55.2	339.22 ± 39.53
V2	331.13 ± 29.56	257.22 ± 46.80**	325.38 ± 35.13	281 ± 42.27	362.63 ± 67	298.78 ± 62.48	384.88 ± 55.32	310.56 ± 47.85*
V3	272.88 ± 35.65	249.89 ± 38.24	300.75 ± 44.75	239 ± 26.52*	328.75 ± 28.13	297.11 ± 65.99	327.25 ± 43.55	270.44 ± 54.81
V4	313.75 ± 29.7	239.78 ± 61.4**	298.87 ± 22.6	232.33 ± 58.92**	350.5 ± 33.4	291.44 ± 51.36	337.88 ± 56.93	243.89 ± 62.09**
V5	308.25 ± 34.78	249.56 ± 49.23*	292.5 ± 24.6	237.22 ± 57.56*	321.25 ± 17.91	267.11 ± 40.87	322.75 ± 37.98	250.11 ± 64.27*

*Note:* *Indicates a statistically significant difference within the same group compared to V1 (*p* < 0.05); **Indicates a statistically significant difference within the same group compared to V1. *p < 0.05; **p < 0.01.

#### 3.4.3 Comparison of Stratum Corneum Hydration

**FIGURE 6:**
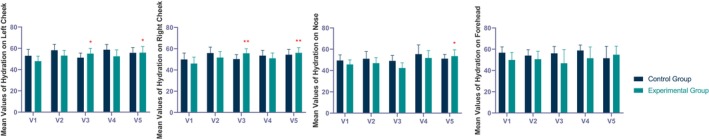
Changes of skin hydration before and after 4 treatments. *, **Indicate a statistically significant difference within the same group compared to V1. *p < 0.05; **p < 0.01.

The hydration of the stratum corneum on both cheeks showed significant improvement in the experimental group compared to the control group at V3 and V5 (*p* < 0.05). At V5, a significant increase in nasal hydration was observed in the experimental group compared to the control group (*p* < 0.05). No noticeable changes were seen in the forehead hydration levels before and after treatment or between the two groups, with no statistical difference (*p* > 0.05). There was no statistically significant difference when comparing each visit time point with the baseline (Table 7 and Figure 6).

**TABLE 7:** Changes of skin hydration before and after 4 treatments (mean ± SD).

Corneometer (a.u.)								
Visits	Left cheek		Right cheek		Nose		Forehead	
	Control group (*n* = 8)	Experimental group (*n* = 9)	Control group (*n* = 8)	Experimental group (*n* = 9)	Control group (*n* = 8)	Experimental group (*n* = 9)	Control group (*n* = 8)	Experimental group (*n* = 9)
V1	52.98 ± 6.01	47.87 ± 4.79	49.92 ± 6.01	46.03 ± 6.01	45.67 ± 4.39	49.46 ± 5.24	56.71 ± 2.74	49.87 ± 3.56
V2	58.16 ± 5.61	53.07 ± 4.96	55.88 ± 5.61	51.71 ± 5.61	46.91 ± 5.22	51 ± 6.93	53.97 ± 2.76	50.54 ± 3.75
V3	51.19 ± 4.27	54.97 ± 4.99*	50.23 ± 4.27	55.67 ± 4.27**	42.38 ± 74.84	49.02 ± 5.23	56.13 ± 3.26	46.79 ± 6.43
V4	58.65 ± 4.97	52.43 ± 6.11	53.51 ± 4.97	50.97 ± 4.97	51.78 ± 7.01	55.35 ± 8.72	58.79 ± 2.55	51.51 ± 5.28
V5	55.72 ± 4.96	55.9 ± 5.86*	54.39 ± 4.96	56.08 ± 4.96**	53.47 ± 6.06	51.17 ± 3.95*	51.51 ± 5.61	54.86 ± 4.03

*Note:* *, **Indicate a statistically significant difference within the same group compared to V1. *p < 0.05; **p < 0.01.

#### 3.4.4 pH Value Changes in the Experimental and Control Groups

**FIGURE 7:**
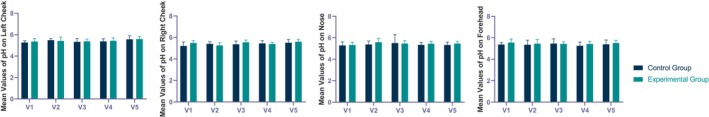
Changes of skin pH before and after 4 treatments.

The pH values of both cheeks generally showed an upward trend in both groups, but no statistically significant difference was found (*p* > 0.05), nor was there any difference within the groups (*p* > 0.05). No differences were observed in nose and forehead at each visit (Table 8 and Figure 7).

**TABLE 8:** Changes of skin pH before and after 4 treatments (mean ± SD).

pH								
Visits	Left cheek		Right cheek		Nose		Forehead	
	Control group (*n* = 8)	Experimental group (*n* = 9)	Control group (*n* = 8)	Experimental group (*n* = 9)	Control group (*n* = 8)	Experimental group (*n* = 9)	Control group (*n* = 8)	Experimental group (*n* = 9)
V1	5.27 ± 0.17	5.36 ± 0.28	5.21 ± 0.36	5.49 ± 0.21	5.28 ± 0.33	5.32 ± 0.26	5.37 ± 0.19	5.55 ± 0.34
V2	5.49 ± 0.16	5.42 ± 0.36	5.4 ± 0.21	5.26 ± 0.24	5.37 ± 0.33	5.58 ± 0.36	5.35 ± 0.43	5.45 ± 0.38
V3	5.33 ± 0.32	5.38 ± 0.21	5.36 ± 0.31	5.55 ± 0.21	5.5 ± 0.8	5.46 ± 0.27	5.46 ± 0.44	5.43 ± 0.19
V4	5.38 ± 0.24	5.43 ± 0.28	5.44 ± 0.26	5.39 ± 0.16	5.34 ± 0.23	5.44 ± 0.22	5.25 ± 0.35	5.42 ± 0.25
V5	5.57 ± 0.32	5.6 ± 0.25	5.51 ± 0.3	5.6 ± 0.21	5.32 ± 0.27	5.46 ± 0.22	5.39 ± 0.4	5.51 ± 0.26

### 3.5 Observation of Adverse Reactions

In this study, one case in the control group developed noticeable erythema over the entire face 1 h after the first treatment, accompanied by itching and burning pain. The symptoms were relieved with oral antihistamines and the topical application of pimecrolimus cream. The reaction was suspected to be an allergy to either the supramolecular salicylic acid simulated product (placebo) or the Broda cold compress used after chemical peeling. In the experimental group, all 15 subjects reported only a negligible burning sensation, and one reported a slightly stinging sensation, which was tolerable and did not interfere with the treatment process.

## 4 Discussion

Rosacea is a chronic inflammatory skin condition with a global prevalence of up to 5.5% [22] and a prevalence of 3.84% [23] prevalence in China. It predominantly affects sebaceous‐rich areas of the face, manifesting with facial erythema, papules, pustules, and telangiectasia, all of which can significantly impact patients' quality of life [23]. The pathophysiology of rosacea is complex, involving impaired skin barrier function [6], elevated TEWL, decreased stratum corneum hydration, and increased skin pH. These alterations contribute to inflammation, forming a vicious cycle that exacerbates the condition. In summary, controlling of inflammation and restoring of the skin barrier are essential strategies in the treatment of rosacea.

Salicylic acid, a well‐known BHA, is both lipophilic and keratolytic, enabling it to penetrate stratum corneum, dissolve excessive sebum [24], and regulate keratinocyte differentiation [25, 26]. Furthermore, SA exhibits strong anti‐inflammatory properties, making it a common treatment for various inflammatory skin conditions and contributing to overall skin health. In previous studies [27, 28, 29] and our clinical experience with low‐concentration SA treatment, we have observed that SA can achieve efficacy comparable to adapalene for some patients with mild to moderate acne. Therefore, we hypothesize that SA may be effective in rosacea, especially on papulopustular subtype. However, like any other chemical peeling agents, SA can also cause irritation during treatment, especially in patients with already compromised skin barrier, due to its keratolytic ability.

Supramolecular salicylic acid is a novel formulation of salicylic acid. By utilizing supramolecular technology, SA is covalently bound to the water‐soluble polymer, poloxamer 407 [15, 16]. This formulation provides SSA a more stable structure as well as the ability to dissolve in water [30]. SSA can remain stable and persistent on the skin, releasing active ingredients slowly and steadily, thereby enhancing the bioavailability of salicylic acid [31] as well as reducing irritation and sting sensations during treatment. This sustained‐release mechanism is especially beneficial for patients with rosacea, who are more susceptible to stimulations from conventional chemical peeling agents. In recent years, SSA has been increasingly used to treat PPR. Studies by Wang et al. [18] combined 30% SSA with minocycline found that the combination therapy was quite effective in 12 weeks. Xu et al. [1] compared the effects of metronidazole gel alone and in combination with 30% SSA in the treatment of PPR and found that the combination therapy was more effective than metronidazole gel alone. Effective management of 30% SSA in combination with other medications for rosacea has been demonstrated, but there has been limited research on 30% SSA as a stand‐alone therapy, and the effects of supramolecular salicylic acid on the facial skin barrier of patients with rosacea require further investigation.

Our study further confirmed that 30% salicylic acid significantly reduces lesion counts and effectively improves persistent erythema in rosacea patients, consistent with previous findings. We did not observe any effects on lesions reduction until 2 weeks after the second treatment (V3). This delayed improvement may be attributed to the nature of chemical peeling agents, where the initial treatment primarily sheds superficial keratin layers without immediately addressing deeper inflammatory papules. Therefore, managing patients' expectations during the early stages of treatment is essential to achieve optimal therapeutic outcomes.

In terms of skin barrier function, our findings are consistent with previous research [18], showing a significant reduction in sebum levels, particularly on the cheeks and forehead, as well as improvements in stratum corneum hydration. However, no significant changes in TEWL or skin pH value, indicating that SA has limited impacts on these parameters.

As the primary action of SA is lipid dissolution, skin sebum was a primary concern in this trial. Our study produced similar results to those of Xu et al. [1], as salicylic acid significantly decreased sebum levels, particularly on the cheeks and forehead. However, no statistically significant improvement was observed in the nose region. We hypothesize that this is due to the standard requirements of chemical peel treatment methods themselves. Because of the thinner and more sensitive skin around the nose, vaseline is required on those area during chemical peeling treatment, restricting the effective area of 30% SSA on nose. Besides, 30% SSA needs physician to massage on patients' skin, the use of rubber gloves may interfere with the procedure on delicate anatomical areas. The application method of 30% SSA chemical peel needs to be improved to have better effectiveness.

SC hydration is an important biophysical parameter in maintaining the skin barrier function [32]. In patients with rosacea, chronic inflammation often leads to impaired keratinocyte function, reducing the ability to maintain normal hydration levels. Notably, unlike Wang's study [18], our study showed not only a downward trend but also a significant increasing in stratum corneum hydration on both cheeks and nose, particularly after the second treatment session. The anti‐inflammatory properties of 30% SSA were considered to contribute to this effect, helping promoting healthier keratinocyte function.

Though topical application of SSA is widely recognized as an effective treatment for various dermatological conditions, its potential adverse effects warrant attention. Previous case reports have indicated that high concentration and extensive topical use of SA (85%–90% area) [33] may lead to excessive systemic absorption, resulting in clinical symptoms such as dizziness, nausea, tinnitus, and hyperventilation [33, 34]. A study evaluated the safety of dermal application of a 30% SA skin peel preparation and found no harm to systemic health [35]. However, similar to common cosmetic products, adverse reactions such as rash, pruritus, and burning sensations due to allergy or irritation during or after treatment are relatively common.

In general, when prescribing 30% SSA, physicians need to thoroughly review the patient's medical history. Particular caution is advised in patients who are systemically using aspirin or other salicylate‐containing medications. Additionally, although SSA has been formulated using supramolecular technology to enhance its gentleness during treatment, the 30% concentration still carries a risk of over‐exfoliation, which may cause adverse effects. Careful monitoring of the treatment endpoint throughout the course of therapy is necessary.

Although the experiment yielded promising results, several limitations should be noted. First, rosacea is a multifactorial condition, and the study design would have benefited from incorporating additional parameters, such as serum analysis, skin scaling, biopsies, and noninvasive techniques like infrared confocal imaging. These measures could complement the VISIA redness index and provide a more comprehensive evaluation of effectiveness of the 30% SSA in controlling inflammation. Second, due to the high recurrence rate of rosacea [36], the relatively short follow‐up period of only 2 weeks posttreatment may not fully capture the long‐term effects. In future studies, we plan to address these limitations by incorporating more extensive data collection and conducting deeper investigations into the treatment of rosacea.

## 5 Conclusion

30% SSA offers a promising treatment for PPR, effectively reducing lesions, improving erythema, reducing sebum, and enhancing skin hydration. However, skin TEWL and pH value show no significant changes. Further studies are needed to explore the long‐term benefits of 30% SSA.

## Author Contributions

Conceptualization: Li Zhang and Xinghua Gao; Methodology: Li Zhang; Formal analysis and investigation: Zihan Wang, Yutong Wu, Xian Su and Fatemeh Nozzari Varkani; Writing original draft preparation: Zihan Wang; Writing – review and editing: Zihan Wang, Li Zhang; Funding acquisition: Li Zhang and Xinghua Gao; Resources: Fatemeh Nozzari Varkani; Supervision: Li Zhang and Xinghua Gao.

## Ethics Statement

This study was conducted in accordance with the Declaration of Helsinki. Ethical approval was obtained from the Ethics Committee of the First Hospital of China Medical University (approval number: [2020‐228]). All participants provided written informed consent before participation.

## Conflicts of Interest

The authors declare no conflicts of interest.

## Data Availability

The data that support the findings of this study are available from the corresponding author upon reasonable request.
